# Development and validation of the Student Social Resilience Scale for Disasters (SSRD) among adolescents in disaster-prone areas

**DOI:** 10.1186/s40359-025-03887-3

**Published:** 2026-01-06

**Authors:** Novia Zalmita, Abdul Manaf, Hizir Sofyan, Mirza Desfandi

**Affiliations:** 1 Graduate School of Social Science Education, Universitas Syiah Kuala, Jl. Tgk. Hasan Krueng Kalee, Darussalam, Banda Aceh, 24415 Indonesia; 2 Department of Geography Education, Universitas Syiah Kuala, Banda Aceh, Indonesia; 3 Research Center for Education, National Research and Innovation Agency, Jakarta, Indonesia; 4 Department of Statistics, Universitas Syiah Kuala, Banda Aceh, Indonesia

**Keywords:** Social resilience, Disaster education, High school students, Scale development, Psychometric validation

## Abstract

The objective of this study is to develop and validate the Student Social Resilience Scale for Disasters (SSRD) as a reliable instrument for measuring the social resilience of high school students in disaster-prone areas. Despite the growing global focus on the issue of resilience, there remains a paucity of instruments that are specifically capable of capturing the multidimensional nature of adolescent social resilience in the context of education, particularly in disaster-affected areas. The present study involved 800 students from high schools in Indonesia and employed Exploratory Factor Analysis (EFA) and Confirmatory Factor Analysis (CFA) to assess the psychometric quality of the SSRD. The EFA results identified three main dimensions, namely Individual, Relationship, and Contextual, while CFA confirmed the construct validity with excellent model fit indices (CFI = 0.968; TLI = 0.965; RMSEA = 0.055). Reliability analysis demonstrated high internal consistency across all dimensions (Cronbach's Alpha 0.925). Moreover, the second-order confirmatory factor analysis (CFA) supported the existence of a second-order construct in the individual factor. This construct was found to consist of self-efficacy and emotional regulation, problem-solving and adaptability, and motivation and perseverance. These findings confirm that SSRD is a valid and reliable instrument for measuring students' social resilience in disaster-prone contexts. In addition to its contribution to the development of theory through the integration of personal, relational, and contextual dimensions, this instrument also has practical implications for education policymakers and practitioners in the design of more targeted disaster education interventions and psychosocial support.

## Introduction

Students in disaster-prone areas need a high level of social resilience to deal with the psychosocial impacts of disasters. Social adaptability helps students recover and remain engaged in learning. However, communities often have low social resilience, making disaster risk reduction challenging [1–3]. Many students experienced difficulties in building supportive relationships after a disaster in schools in disaster-prone areas, such as Indonesia [4, 5]. Furthermore, the development of measurement tools designed specifically for adolescents in regions characterised by distinct socio-cultural and risk of disaster profiles, such as Indonesia, remains limited [6, 7]. This indicates the need for a measurement tool to identify students' level of social resilience. This study aims to develop and validate the Student Social Resilience Scale for Disasters (SSRD) to support educational interventions in disaster-prone schools.

In the literature, social resilience is defined as the ability of communities to withstand, adapt to and recover from crises, including natural disasters [8, 9]. This concept is multidimensional, covering social, economic, cultural, institutional, physical and human aspects [10, 11]. High school students as adolescents are in a vulnerable phase of development but are important in shaping social adaptability [12]. Support from peers and family plays a major role in building students' resilience [13], which contributes to increased resilience [14]. However, most studies on social resilience still focus on post-disaster communities [15, 16] or adult groups [17, 18], so measuring social resilience in high school adolescents is still very rare.

Resilience is a key protective factor for individuals experiencing disasters because it helps them cope with traumatic events, reduces levels of depression and anxiety, and improves their ability to adapt to various difficulties. Therefore, disaster education needs to be directed at strengthening psychological aspects such as self-efficacy and self-confidence as an important foundation for building personal resilience [19]. In addition, resilience is not only determined by individual capacity but is also influenced by contextual factors that include community participation, access to resources, and institutional support. These three aspects play an important role in shaping community-based social resilience, where communities can collaborate, support each other, and effectively utilize local resources to deal with disaster risks in a sustainable manner [20].

Social resilience among secondary school adolescents is a strategic issue in global disaster risk management, especially in disaster-prone areas that often experience post-disaster psychosocial distress [21]. Despite the existence of numerous resilience measurement tools on the international stage, most of these tools are characterized by their general nature, their focus on adult populations, or their inability to comprehensively capture the multifaceted dynamics of social adaptation within the school environment. Recent literature indicates that social-cultural contexts, school structures, and local disaster experiences significantly influence the construction of resilience [22, 23]. This underscores the limitations of prevailing adaptation tools, which frequently fail to consider local factors such as community norms, teacher-student relationships, and school social capital.

The development of SSRD in this study is based on the theoretical framework of the Children and Youth Resilience Measure (CYRM-28), a widely recognised measurement tool for assessing resilience through three core dimensions: Individual Factors, Relationships with Primary Caregivers, and Contextual Factors [24]. The CYRM-28 is predicated on a socio-ecological resilience framework, which posits that resilience emanates from the interaction between personal strengths, supportive relationships, and environmental resources. This framework aligns with the conditions experienced by adolescents in disaster-prone areas, where resilience is shaped not only by individual capacity but also by family support, peer networks, and the broader community system [12, 13]. However, despite its strong theoretical basis, CYRM-28 was developed for the adolescent population in general and does not specifically capture the social dynamics related to disasters in the school environment. Consequently, CYRM-28 functions as a conceptual basis for SSRD, which adapts and expands its scope to encompass the distinctive social processes that influence student resilience within the context of disasters.

A comparison of the SSRD with existing resilience instruments highlights its novelty and contextual relevance. The CYRM-28 [24] is a multifaceted instrument that assesses individual, relational, and contextual dimensions; however, it is deficient in its lack of specificity for school disaster contexts and its failure to measure school social capital. The Multidimensional Scale of Perceived Social Support (MSPSS) [25] focuses exclusively on support from family, friends, and significant others, thereby neglecting to capture resilience as a multidimensional construct. In a similar vein, the Resilience Scale [7] places significant emphasis on personal competence and self-acceptance. However, its relevance to school-aged adolescents is limited, as it fails to consider teacher–student relationships and the broader educational ecology. Conversely, the SSRD, a framework developed for high school students in disaster-prone regions, integrates individual, relational, and school-based contextual factors, thereby addressing a significant gap in resilience measurement within school-based disaster contexts.

This paper aims to create novelty by integrating the social resilience of high school students in the context of disaster resilience in vulnerable areas. The focus on high school students as a critical age group is also unique, given that their cognitive and emotional developmental stages are crucial in building disaster mitigation awareness [26, 27]. In addition, this study developed a Likert scale specifically to measure the social resilience of high school students, ensuring the relevance of the measurement tool to the population under study. Using a quantitative approach, this study also explores the social dimensions of disaster resilience, such as support from peers, family and school, which play an important role in shaping students' resilience [14].

The dearth of measurement tools specifically designed to identify students' social resilience in disaster-prone areas suggests an urgent need for the development of contextually relevant instruments. Existing instruments are typically generalized, failing to consider the local characteristics, culture, and unique experiences of students in areas, which are often affected by disasters [28]. Consequently, numerous educational assistance or intervention efforts post-disaster are not targeted due to a lack of a profound comprehension of students' social resilience [29]. The SSRD is a valid and reliable instrument that enables educational institutions and policymakers to accurately identify students' social vulnerabilities and strengths. This knowledge facilitates the development of targeted interventions that address the specific needs of the students, thereby enhancing the resilience of the school community [30].

This research is of significant urgency because high school students are regarded as agents of change with considerable potential in establishing disaster-resilient communities. A comprehensive understanding of their social resilience can serve as a foundational element in the development of more effective interventions to enhance the preparedness of young individuals in confronting disasters. Furthermore, this research contributes to the enhancement of education policy through the provision of pragmatic recommendations for the incorporation of disaster awareness programs into the formal curriculum, thereby ensuring the systematic preparation of the younger generation. Moreover, this research enhances the global literature by providing empirical data from developing countries, such as Indonesia, which are frequently underrepresented as disaster-prone regions. This contextual insight provides substantial evidence for the formulation of pertinent risk mitigation strategies, not only for the local context but also for countries facing analogous challenges. This novelty is a critical foundation for understanding the role of the younger generation in building social resilience in disaster-prone areas.

## Research methods

### Participants

The present study was designed to test the Student Social Resilience Scale for Disasters (SSRD) instrument. The sample size was designed to be large-scale, referring to the ideal recommendations for national studies, complex models, and measurement invariance testing, involving ≥ 500 respondents with an optimal target of 800 [31, 32]. The respondents in this study were high school students aged 15–18 years who were enrolled in grade X–XII level courses. The gender composition of the respondents included both male and female students. A substantial sample size was selected to augment the precision of parameter estimates, the stability of the model fit index, and the power of cross-subgroup testing [33]. The sampling technique employed was stratified multistage cluster sampling, wherein the population was segmented into strata based on the nature of the disaster (natural, non-natural, social/conflict). This was followed by a multistage selection process, commencing from administrative regions, schools, and culminating in the selection of students at random [34, 35]. Sampling was conducted in various disaster-affected areas across Indonesia, reflecting the diversity of geographical, social, and cultural conditions [21]. The respondents were high school students residing in disaster-affected regions, thereby ensuring that the data obtained could represent national adolescent social resilience for the development and validation of SSRD.

### Design

The SSRD instrument was developed using a modified method based on the CYRM-28 proposed by Liebenberg et al. [24]. The CYRM-28 was considered suitable due to its ability to measure the social dimensions of resilience, such as support from family and community, which are essential for adolescents in disaster settings [24, 36, 37]. Other experts have also emphasized the use of tools like CYRM-28 that assess resilience through individual, relational, and contextual aspects. This perspective highlights that children's adaptation success depends on their interactions with and the quality of services in their environment [20]. The original CYRM-28 statements were adapted to create the SSRD, with a focus on measuring students’ social resilience in disaster contexts.

### Measures, item and scale development

In this study, the SSRD was developed by the researcher based on the CYRM-28 instrument, with several modifications and adjustments to the original statements. This new scale was designed to meet the specific needs of measuring the social resilience of high school students in facing disasters. The CYRM-28 instrument developed by Liebenberg et al. [24] consists of three main dimensions: Individual Factors, Relationships with Primary Caregivers, and Contextual Factors, comprising 28 statement items. In the SSRD, these items were adapted and revised so that the statements more directly reflect students’ social resilience in disaster contexts. The final version of the SSRD, presented in Table 1, uses a five-point Likert scale (Often, Somewhat Often, Sometimes, Rarely, Not at All) [24, 36, 37].

**Table 1:** Items of Student Social Resilience Scale for Disasters (SSRD)

Original Item*	Forward (Indonesian)	Final item (Indonesian — after translation and review)	Forward (English)	No Item
I cooperate with people around me	Saya menjalin kerja sama yang baik dengan orang-orang di sekitar saya	saya menjalin hubungan kerja sama yang kuat dengan tetangga dan komunitas untuk mempersiapkan diri menghadapi kemungkinan darurat dan saling membantu	I establish strong working relationships with neighbors and the community to prepare for emergencies and help each other	1
I aim to finish what I start	Saya bertujuan untuk menyelesaikan apa yang saya mulai	Sebelum terjadi bencana, saya selalu bertekad menyelesaikan segala persiapan dan tindakan pencegahan, penyelamatan dan pemulihan hingga tuntas	Before a disaster strikes, I am always determined to complete all preparations and preventive, rescue, and recovery measures thoroughly	2
I solve problems without drugs or alcohol	Saya memecahkan masalah tanpa narkoba atau alkohol	saya memilih cara yang sehat dan positif untuk menghadapi dan mengatasi tekanan	I choose healthy and positive ways to deal with and overcome stress	4
I am aware of my own strengths	Saya menyadari kekuatan saya sendiri	saya menyadari kekuatan dan kemampuan diri dalam bersiap menghadapi situasi darurat	I am aware of my strengths and abilities in preparing for emergency situations	5
I have people I look up to	Saya memiliki orang-orang yang saya hormati	saya menghormati pemimpin komunitas dan para pekerja kemanusiaan yang bertugas	I respect community leaders and humanitarian workers	19
I feel supported by my friends	Saya merasa didukung oleh teman-teman	saya merasa didukung oleh teman-teman dalam menghadapi kemungkinan darurat	I feel supported by my friends in facing potential emergencies	8
My friends stand by me during difficult times	Teman-teman saya selalu ada di samping saya saat masa-masa sulit	teman-teman saya selalu siap membantu dan bersama saya dalam keadaan darurat	My friends are always ready to help and be with me in emergencies	9
People think I am fun to be with	Orang-orang menganggap saya menyenangkan untuk diajak bergaul	saya dianggap sebagai orang yang positif dan menyenangkan, sehingga mudah menjalin hubungan dengan komunitas	I am considered a positive and pleasant person, so it is easy for me to build relationships with the community	3
I know how to behave in different social situations	Saya tahu bagaimana berperilaku dalam berbagai situasi sosial	saya sudah memiliki keterampilan sosial yang baik, sehingga dapat beradaptasi dalam berbagai situasi darurat	I have good social skills, so I can adapt to various emergency situations	20
I am given opportunities to become an adult	Saya diberi kesempatan untuk menjadi dewasa	pengalaman dalam menghadapi bencana membantu saya tumbuh dan menjadi pribadi yang lebih dewasa	My experience in dealing with disasters has helped me grow and become a more mature person	21
I know where to go to get help	Saya tahu ke mana harus mencari bantuan	saya mengetahui sumber daya dan lembaga yang bisa diandalkan untuk mencari bantuan	I know reliable resources and institutions where I can seek help	22
I have opportunities to develop job skills	Saya memiliki kesempatan untuk mengembangkan keterampilan kerja	saya memiliki kesempatan untuk mengembangkan keterampilan yang berguna, termasuk keterampilan tanggap darurat	I have the opportunity to develop useful skills, including emergency response skills	23
I eat enough most days	Saya makan cukup hampir setiap hari	Dalam kondisi darurat, saya memiliki akses yang baik terhadap makanan dan menjaga asupan nutrisi sehari-hari	In an emergency, I have good access to food and maintain my daily nutritional intake	12
I feel safe when I am with my caregivers	Saya merasa aman ketika bersama keluarga/wali saya	saya selalu merasa aman dan nyaman berada di dekat keluarga atau wali saya	I always feel safe and comfortable being close to my family or caregivers	15
My caregivers watch me closely	Keluarga/wali saya mengawasi saya dengan seksama	keluarga atau wali saya selalu memperhatikan kesejahteraan dan keselamatan saya dengan baik	My family or caregivers always pay close attention to my well-being and safety	10
My caregivers know a lot about me	Keluarga/wali saya tahu banyak tentang saya	keluarga atau wali saya memahami kebutuhan, kondisi, dan perasaan saya dengan baik	My family or caregivers understand my needs, circumstances, and feelings well	11
I talk to my caregivers about how I feel	Saya mengutarakan kepada keluarga/wali saya tentang apa yang saya rasakan	saya selalu terbuka kepada keluarga atau wali mengenai perasaan dan kecemasan yang saya alami	I am always open with my family or caregivers about my feelings and concerns	13
My caregivers stand by me during difficult times	Keluarga/wali menemani saya di saat sulit (misalnya ketika saya sakit, kebingungan, dsb)	keluarga atau wali saya selalu hadir untuk mendampingi saya dalam masa-masa sulit atau ketika saya membutuhkan dukungan	My family or caregivers are always there for me during difficult times or when I need support	14
I enjoy my caregivers’ cultural and family traditions	Saya menyukai tradisi dan kebiasaan keluarga/wali saya	Tradisi dan kebiasaan keluarga atau wali saya memberi saya rasa nyaman dan aman	My family or caregivers’ traditions and customs give me a sense of comfort and security	16
Spiritual beliefs are a source of strength for me	Kepercayaan spiritual merupakan sumber kekuatan bagi saya	keyakinan spiritual saya menjadi sumber kekuatan dalam menjalani kehidupan sehari-hari dan memberikan kekuatan menghadapi kemungkinan darurat	My spiritual beliefs are a source of strength in my daily life and give me the strength to face emergencies	6
I participate in organized religious activities	Saya berpartisipasi dalam kegiatan keagamaan yang terorganisasi	saya terlibat dalam kegiatan keagamaan yang memberikan dukungan moral dan spiritual bagi saya	I participate in religious activities that provide me with moral and spiritual support	26
Getting an education is important to me	Mendapatkan pendidikan adalah hal penting bagi saya	saya menempatkan pendidikan sebagai prioritas untuk masa depan yang lebih baik, termasuk pengetahuan tentang kesiapsiagaan bencana	I prioritize education for a better future, including knowledge about disaster preparedness	17
I feel I belong at my school	Saya merasa diterima di sekolah saya	saya merasa diterima di sekolah dan berpartisipasi aktif dalam kegiatan belajar serta program kesiapsiagaan	I feel accepted at school and actively participate in learning activities and preparedness programs	18
I think it is important to serve my community	Saya pikir penting untuk melayani komunitas saya	saya aktif dalam kegiatan sosial dan pelayanan untuk membantu komunitas dalam pencegahan dan kesiapsiagaan	I am active in social and service activities to help the community in prevention and preparedness	7
I am proud of my ethnic background	Saya bangga dengan latar belakang etnis saya	saya bangga dengan latar belakang etnis saya yang mengajarkan nilai-nilai kekuatan dan solidaritas dalam menghadapi tantangan	I am proud of my ethnic background, which teaches me the values of strength and solidarity in the face of challenges	24
I am treated fairly in my community	Saya diperlakukan adil di dalam kelompok/komunitas saya	saya merasa bahwa komunitas saya memperlakukan semua anggotanya dengan adil dan setara	I feel that my community treats all its members fairly and equally	25
I enjoy my community’s traditions	Saya menikmati tradisi komunitas saya	Tradisi komunitas saya memberikan rasa persatuan dan kekuatan yang membantu dalam menghadapi kondisi darurat	My community's traditions provide a sense of unity and strength that helps in dealing with emergencies	27
I am proud of my citizenship	Saya bangga dengan kewarganegaraan saya	saya bangga dengan kewarganegaraan saya yang berperan dalam mempersiapkan warganya menghadapi kemungkinan darurat dan bencana	I am proud of my citizenship, which plays a role in preparing its citizens for possible emergencies and disasters	28

^*^original items Copyright © 2018 Liebenberg et al., 2012

Table 1 presents the statement items in the SSRD, which was adapted from the CYRM-28 scale. The development process is carried out through translation, concept review, and adjustment to the socio-cultural context of secondary school students in disaster-prone areas. Each original item in English was translated into Indonesian, then substantively adjusted to reflect local values, disaster experiences, and students' social engagement. For instance, the statement "I cooperate with people around me" was modified to "I establish strong cooperative relationships with neighbors and communities to prepare for possible emergencies," which is more contextual to disaster situations. This development indicates that the instrument underwent not only literal translation but also conceptual development to encompass aspects of social resilience in a holistic manner, incorporating individual, relational, spiritual, and community dimensions within the framework of disaster preparedness.

The instrument adaptation process involves the instrument development step, which entails the modification of various elements to align more closely with students' experiences of disasters. This process encompasses adjustments to linguistic aspects and the reinforcement of conceptual constructs, ensuring that the instrument is capable of comprehensively capturing the individual, relational, spiritual, and community dimensions. To ensure the readability and clarity of the items, a pilot test was conducted on a small group of high school students (n = 30). The evaluation process centered on assessing language comprehension, the clarity of statements, and the relevance of the disaster context. Preliminary findings from the pilot test guided the implementation of numerous refinements, which were subsequently integrated into the final iteration of the SSRD employed in this study (see Table 1).

To ensure that the instrument was valid and consistent, several psychometric procedures were conducted on the SSRD. In this study, Exploratory Factor Analysis (EFA) was used to identify and explore the underlying factor structure and to determine the indicators most appropriate for measuring the intended constructs [38, 39]. The application of EFA was essential because a previously validated factor structure cannot be assumed to hold across different populations or contexts. Variations in culture, language, educational settings, and respondent characteristics may alter item functioning and the relationships among indicators, necessitating empirical verification of the construct structure before subsequent analyses are performed. This aligns with psychometric guidelines stating that construct validity is sample-dependent, and that any instrument adapted for use in a new population requires an empirical evaluation of its factor structure through EFA [40, 41].

Following the establishment of the factor structure, internal consistency was examined using Cronbach’s Alpha to ensure that each indicator within the extracted factors demonstrated an acceptable level of reliability [42]. Subsequently, Confirmatory Factor Analysis (CFA) was employed to confirm and validate the measurement model derived from the EFA, thereby assessing the model’s overall goodness-of-fit with the data [38, 43]. All analyses, including EFA, Cronbach’s Alpha, and CFA, were performed using Jeffrey's Amazing Statistics Program (JASP) version 0.19.3 [44].

## Results

### Exploratory Factor Analysis (EFA)

Prior to conducting a data analysis using EFA, a preliminary data check was performed using the Kaiser–Meyer–Olkin (KMO) and Bartlett's Test. These two tests are used to evaluate the suitability of data for EFA [45, 46]. The results of the KMO Test indicated an Overall Measure of Sampling Adequacy (MSA) value of 0.911, which is regarded as excellent according to the KMO interpretation. This finding suggests that the available data is adequate for conducting factor analysis. Furthermore, the KMO values for each variable (individual MSA) are all above 0.8, with most variables having values close to or greater than 0.9. This finding suggests that each variable possesses sufficient correlation with the other variables to facilitate factor analysis. The analysis revealed that no variable possesses an MSA value below 0.5, thereby ensuring that no variables must be excluded from the subsequent analysis. Moreover, the results of Bartlett's Test indicate a chi-square (X^2^) value of 10,696.904 with degrees of freedom (df) of 378, and a significance value of *p* < 0.001. Given the substantial outcomes of Bartlett's Test and the prior elevated KMO value, the data is deemed appropriate for advancing to EFA.

EFA using the varimax rotation method (see Table 2) identified three main factors consistent with the theoretical construct of the SSRD: individual, relationship, and contextual. The individual factor includes most of the items (e.g., V1, V2, V5, V7, V20, V23, and V27) with loading values ranging from 0.351 to 0.719. These items represent personal capacity for coping with disasters. The Relationship factor includes items such as V10, V13, V14, V15, and V16, which have loading values ranging from 0.316 to 0.851 and reflect social support and the quality of interactions. The contextual factor includes items such as V8 and V9, which have loadings ranging from 0.630 to 0.772 and describe environmental support and community resources. Uniqueness values are mostly below 0.70, indicating that the model adequately explains the variance. However, some items (V3, V19, V21, and V26) have low contributions, yet they can still be used because they fall within the recommended loading values. These findings align with methodological guidelines that recommend retaining items with loadings of at least 0.30–0.40 for stable factor models [32, 33].

**Table 2:** EFA results

Item	Individual	Relationship	Contextual	Uniqueness
V1	0.489			0.657
V2	0.554			0.668
V3	0.422			0.746
V4	0.493			0.684
V5	0.611			0.613
V6	0.511			0.663
V7	0.549			0.665
V8	0.351		0.630	0.410
V9		0.327	0.772	0.243
V10		0.616		0.584
V11		0.655		0.481
V12	0.489	0.344		0.597
V13		0.639		0.489
V14		0.813		0.281
V15		0.776		0.379
V16		0.851		0.235
V17	0.493			0.666
V18	0.495		0.311	0.600
V19	0.396		0.346	0.723
V20	0.678			0.496
V21	0.405			0.765
V22	0.640			0.565
V23	0.719			0.430
V24	0.543			0.651
V25	0.513	0.316		0.597
V26	0.457			0.767
V27	0.643			0.501
V28	0.510			0.638

Applied rotation method is varimax

### Relialibility (Cronbach’s Alpha)

In this study, reliability was measured as internal consistency among items in the SSRD instrument. Reliability testing is important to ensure that the obtained data accurately reflects the measured constructs in a stable and reliable manner. A reliable instrument produces consistent measurement results not only at one time, but also when reused under similar conditions. In instrument development research, reliability also indicates that each statement has a logical and homogeneous relationship with the overall construct. Therefore, reliability testing is a crucial stage in instrument validation. In this study, it was carried out using Cronbach's alpha to measure the extent to which items in each dimension of the instrument are related and consistent.

As demonstrated in Table 3, the results of the reliability analysis indicate that the SSRD instrument exhibits excellent internal consistency. McDonald's Omega (ω = 0.925), Cronbach's Alpha (α = 0.925), and Guttman's λ2 (0.927) values all exceed the threshold of 0.90, indicating very high reliability. The 95% confidence interval is relatively narrow (e.g., α = 0.917–0.933), thus indicating that the estimates are stable and precise across a range of possible samples. Furthermore, the average inter-item correlation value of 0.306 falls within the optimal range of 0.20–0.40, thereby suggesting a moderate correlation between items and thereby substantiating the multidimensional character of the construct under scrutiny. The findings of this study demonstrate the consistency and collaborative functionality of the SSRD items in measuring social resilience, thereby providing substantial empirical evidence for the reliability of the instrument within the context of schools in disaster-prone areas [32, 47, 48].

**Table 3:** SSRD reliability results

*Frequentist Scale Reliability Statistics*					
				95% CI	
Coefficient	Estimate		Std. Error	Lower	Upper
Coefficient ω	0.925		0.004	0.918	0.933
Coefficient α	0.925		0.004	0.917	0.933
Guttman's λ2	0.927		0.004	0.919	0.934
Average interitem correlation	0.306				
Mean	109.617		0.619	108.404	110.830
Variance	306.727		15.336	278.755	339.153
SD	17.514		0.424	16.696	18.416

The standard error of the average interitem correlation is not available

### Confirmatory Factor Analysis (CFA)

CFA was conducted to evaluate the factor structure that emerged from the preceding EFA. The present approach was selected on account of the fact that SSRD underwent substantial contextual modifications from the original version of CYRM-28. Consequently, empirical exploration of the underlying dimensions was deemed necessary prior to confirmation. The CFA results demonstrated that the exploratory factor model generated from EFA exhibited a strong fit with the observed data, as evidenced by various fit index indicators and fit measures.alignment with the data, as evidenced by numerous indicators of fit indices and fit measures.

According to the model fit results in Table 4, the results of the chi-squared test demonstrate a value of χ^2^ (344) = 1168.969, *p* < 0.001. A *p*-value of less than 0.001 indicates a significant difference between the tested model and the good fit model [49]. However, it is important to note that the Chi-Square test is highly sensitive to large sample sizes. Therefore, it is essential that model interpretation does not rely solely on this value. Consequently, the utilization of supplementary evaluation employing additional fit indices, such as Comparative Fit Index (CFI), Tucker-Lewis Index (TLI), Root Mean Square Error of Approximation (RMSEA), and Standardized Root Mean Square Residual (SRMR), is imperative to obtain a more comprehensive representation of the model's compatibility with the data.

**Table 4:** Model fit (Indices and measures) summary statistics of SSRD confirmatory factor analysis

Model Fit	Fit Index	Value	Cut-off Criteria
Chi-Square test	Chi-squared χ2	1168.969	χ^2^/df < 2 (excellent fit)
	df	344	
	p	<.001	p < 0.05
Fit Indices	Comparative Fit Index (CFI)	0.968	≥ 0.95 (excellent fit)
	Tucker-Lewis Index (TLI)	0.965	≥ 0.95 (excellent fit)
Fit Measures	Root mean square error of approximation (RMSEA)	0.055	< 0.08 (good fit)
	Standardized root mean square residual (SRMR)	0.063	< 0.08 (good fit)
	Goodness of fit index (GFI)	0.997	≥ 0.95 (excellent fit)
	Hoelter's critical N (α =.05)	266.705	More than 280 (suitable)
	Hoelter's critical N (α =.01)	280.182	

The model demonstrated a very good fit, as indicated by the CFI value of 0.968 and the TLI value of 0.965. The value exceeds the limit of ≥ 0.95, indicating that the model has an excellent fit. Furthermore, the RMSEA of 0.055 with a 90% confidence interval between 0.051 and 0.058 indicates that the model is within acceptable fit limits, with a p-value of 0.012 indicating no significant difference between the model and the population data. Additionally, the SRMR value of 0.063, which is below the threshold of 0.08, indicates that the model has adequate fit. Furthermore, the Goodness of Fit Index (GFI) obtained was 0.997, indicating that the model is capable of explaining the majority of the variance in the data. The analysis further demonstrated that Hoelter's critical N value was 266.705 at a significance level of 0.05 and 280.182 at a significance level of 0.01, indicating that the model exhibited robust stability in large sample sizes. This finding serves to reinforce the structural validity of SSRD for measuring student social resilience in disaster-prone areas [50]. In consideration of the aforementioned results, it can be concluded that the developed model demonstrates a satisfactory degree of fit, thereby indicating its suitability for subsequent analyses aimed at evaluating the factors under evaluation.

The CFA analysis results (Table 5) demonstrate that all indicators exhibit significant factor loadings (*p* < 0.001) with standardized loadings exceeding 0.50, thereby satisfying the criteria for convergent validity [32, 33]. The Individual factor is comprised of 17 indicators, with loadings ranging from 0.548 to 0.797. The indicators that contributed most significantly to the model were V23 (0.797), V27 (0.750), and V25 (0.702), while the indicators with the lowest contributions were V26 (0.548). Although this is relatively low, it still meets the feasibility threshold. The Relationship Factor is comprised of six indicators, with a very strong loading range of 0.785–0.898. The strongest indicators are V16 (0.898) and V14 (0.892). The contextual factor is represented by two indicators, namely V8 (0.881) and V9 (0.861), both of which provide a high level of contribution. The findings of this study corroborate the validity and reliability of the SSRD, which has been demonstrated to possess a three-factor structure encompassing the domains of individual, social relationships, and environmental context. These domains are identified as the primary dimensions of student social resilience within disaster-prone regions [32, 51, 52].

**Table 5:** Factor loadings of confirmatory factor analysis

Factor	Indicator	Std. Estimate	Std. Error	z-value	*p*	95% Confidence Interval	
						Lower	Upper
Individual	V23	0.797	0.026	30.428	<.001	0.746	0.849
	V1	0.648	0.038	16.829	<.001	0.572	0.723
	V2	0.595	0.042	14.139	<.001	0.513	0.678
	V3	0.594	0.041	14.313	<.001	0.513	0.675
	V4	0.626	0.042	14.909	<.001	0.543	0.708
	V5	0.604	0.040	15.103	<.001	0.525	0.682
	V6	0.588	0.041	14.250	<.001	0.507	0.668
	V7	0.634	0.040	15.996	<.001	0.556	0.712
	V12	0.702	0.034	20.709	<.001	0.635	0.768
	V17	0.613	0.043	14.339	<.001	0.529	0.697
	V18	0.672	0.039	17.230	<.001	0.595	0.748
	V19	0.563	0.045	12.538	<.001	0.475	0.651
	V20	0.709	0.036	19.464	<.001	0.638	0.780
	V21	0.618	0.042	14.662	<.001	0.535	0.701
	V22	0.663	0.037	17.894	<.001	0.590	0.735
	V24	0.648	0.038	16.959	<.001	0.573	0.723
	V25	0.702	0.036	19.422	<.001	0.631	0.773
	V26	0.548	0.046	11.795	<.001	0.457	0.639
	V27	0.750	0.031	24.007	<.001	0.689	0.811
	V28	0.686	0.037	18.329	<.001	0.613	0.760
Relationship	V10	0.785	0.037	21.071	<.001	0.712	0.858
	V11	0.841	0.027	31.057	<.001	0.788	0.894
	V13	0.827	0.031	26.370	<.001	0.765	0.888
	V14	0.892	0.020	43.792	<.001	0.852	0.932
	V15	0.847	0.028	30.131	<.001	0.792	0.902
	V16	0.898	0.023	39.174	<.001	0.853	0.943
Contextual	V8	0.881	0.029	30.506	<.001	0.824	0.938
	V9	0.861	0.028	30.337	<.001	0.806	0.917

**Fig. 1:**
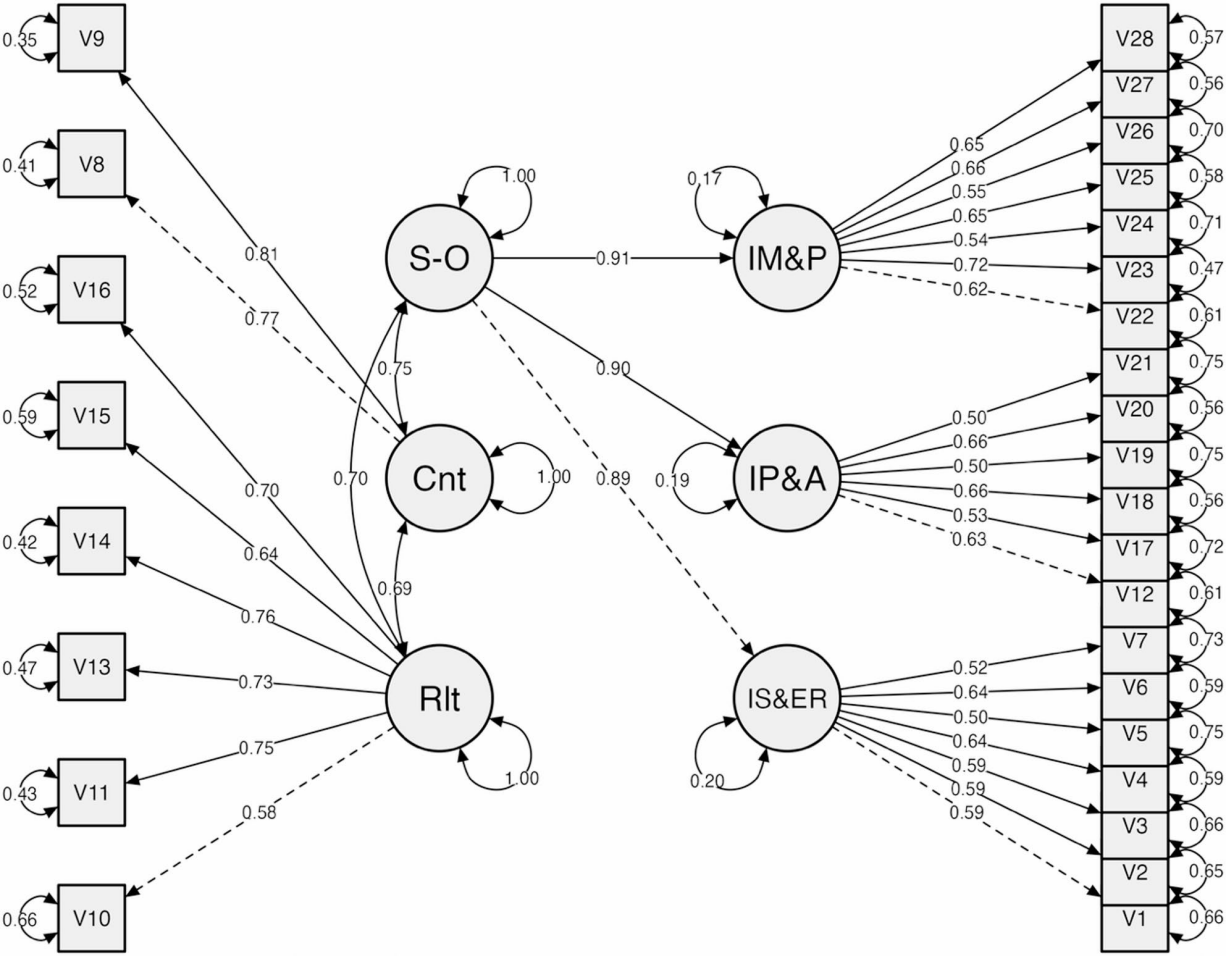
Path diagram Confirmatory Factor Analysis (CFA) of SSRD. *Note*. Cnt = Contextual; Rlt = Relationship; S–O = Second Order; IM&P = Individual Motivation & Perseverance); IP&A = Individual Problem-solving & Adaptability; IS&ER = Individual Self-efficacy & Emotional Regulation

The results of the second-order CFA (see Table 6) demonstrate that the individual factor is divided into three subdimensions, all of which exhibit extremely high and significant factor loadings (*p* < 0.001). The Individual Self-Efficacy and Emotional Regulation subdimension demonstrated a loading of 0.895, the Individual Problem-Solving and Adaptability subdimension exhibited a loading of 0.899, and the Individual Motivation and Perseverance subdimension showed a loading of 0.912. This range of values (0.895–0.912) reflects the very strong contribution of the three subdimensions to the main Individual construct. The findings of this study demonstrate that individual resilience in students is a multidimensional construct that cannot be adequately measured through a single aspect. Rather, it requires the integration of self-regulation abilities, adaptation skills, and motivation and perseverance. Consequently, the implementation of a second-order model in SSRD facilitates the capture of the intricacies inherent in individual factors in a more comprehensive and theoretically consistent manner (Fig. 1).

**Table 6:** Second-order factor loadings of individual factor

						95% Confidence Interval	
Factor	Indicator	Std. estimate	Std. Error	z-value	*p*	Lower	Upper
Second Order	Individual Self-efficacy & Emotional Regulation	0.839	0.025	34.058	<.001	0.791	0.888
	Individual Problem-solving & Adaptability	0.912	0.024	38.576	<.001	0.866	0.959
	Individual Motivation & Perseverance	0.909	0.022	40.569	<.001	0.865	0.953

The second-order CFA model of the SSRD (Fig. 1) demonstrates that student social resilience is formed by three primary dimensions: Individual, Relationship, and Contextual. The Individual dimension is mapped as a second-order factor with three subdimensions, namely Motivation & Perseverance (IM&P), Problem-solving & Adaptability (IP&A), and Self-efficacy & Emotional Regulation (IS&ER). Each of these subdimensions has high loadings (0.89–0.91). The indicators in each dimension also show adequate factor loadings, thus confirming the construct validity. The magnitude of the observed correlations between constructs ranged from moderate to high, indicating a strong conceptual relationship while maintaining the uniqueness of the construct dimensions [11]. The findings of this study serve to substantiate the notion that SSRD is a multifaceted construct that reflects social resilience. The integration of personal strength, relational support, and contextual factors is particularly salient in the context of disaster-prone areas, where they collectively contribute to the shaping of student resilience.

It is noteworthy that the second-order structure observed in the SSRD differs from that of the CYRM-28, where all three dimensions exhibit a relatively uniform second-order configuration. In this study, only the Individual dimension formed a clear second-order structure, while the Relationship and Contextual domains remained first-order factors. This divergence suggests that, within the Indonesian high school context, personal capacities may exert a stronger influence on social resilience than relational or environmental factors—a trend similarly reported in studies involving high-risk youth but less evident in general populations. These findings underscore that instrument adaptation is not merely a linguistic process but also a conceptual one, shaped by the cultural and psychosocial characteristics of the target population. As such, the resulting SSRD structure provides a more contextually grounded and meaningful representation of how students in disaster-prone regions develop social resilience.

## Discussion

The present study developed and validated the SSRD using both exploratory and confirmatory factor analyses, yielding a three-factor structure—Individual, Relationship, and Contextual—that demonstrated strong reliability and validity. The CFA results confirmed that the EFA-derived model achieved excellent fit across several indices, while convergent validity further supported the coherence of the constructs. Although two indicators were removed due to low contribution, the remaining items reflected a holistic conceptualization of students’ social resilience in disaster contexts. These findings are consistent with prior research demonstrating that resilience instruments grounded in social–ecological theory tend to exhibit multidimensional structures among adolescents [7, 19]. However, some studies have reported alternative patterns. For instance, Shi et al. [7] found that resilience among disaster-exposed adolescents clustered primarily around individual competencies rather than contextual dimensions, while other studies have shown a stronger dominance of relational support over individual attributes. These contrasting findings highlight that resilience structures may differ across cultural, developmental, and hazard contexts. Thus, the SSRD contributes to this line of inquiry by offering a measurement model that is specifically responsive to the social and environmental realities of students in disaster-prone Indonesian regions.

The applicability of the SSRD is further reinforced by its relevance to Indonesia’s socio-cultural landscape, which is shaped by recurring natural disasters, political transitions, and uneven economic development. In such contexts, students' resilience is influenced by systemic instability and varying degrees of institutional support. Drawing on Bronfenbrenner’s Social Ecology Theory [53], the interaction between individual capacities, social relationships, and broader contextual systems becomes essential for understanding resilience formation. While the present study supports this multi-layered perspective, it is important to acknowledge that not all research aligns with this view. Some studies report that external structural factors, such as governance quality or economic deprivation, may overshadow the influence of school-based support, whereas others suggest that strong peer and family networks can compensate for structural limitations. These divergent findings indicate that resilience processes are highly context dependent. Indonesia’s unique autonomy framework offers opportunities to integrate disaster education into local school curricula, yet the effectiveness of these initiatives remains contingent upon consistent implementation and resource availability. Therefore, the SSRD emphasizes the need for resilience assessments that account for local cultural, economic, and political conditions—ensuring more contextually grounded interpretations, especially in disaster-prone regions like Indonesia.

In this study, the concept of student social resilience is understood to encompass three primary dimensions: individual, relationship, and contextual. This conceptualisation reflects the notion that resilience is a multidimensional construct. Specifically, the Individual dimension does not stand as a single factor but consists of three important sub-indicators, namely Self-efficacy & Emotional Regulation, Problem-solving & Adaptability, and Motivation & Perseverance, which were identified through second-order CFA analysis. This finding lends further credence to the notion that students' individual resilience is shaped by their capacity to effectively manage emotions, proactively solve problems, and sustain motivation in the face of adversity. These findings align with those of Ungar [29], who posits that resilience is the result of the interaction between individual capacity and social resources, and those of Masten et al. [54], who emphasise the importance of a multisystem approach in understanding the resilience of children and adolescents. Furthermore, Shi et al. [7] emphasise that resilience in disaster-affected adolescents must consider social and environmental factors. This study serves to reinforce the existing body of evidence that the assessment of students' social resilience must encompass personal, relational, and contextual dimensions through the Individual sub-indicator, in order to adequately capture the intricacies of the socio-cultural milieu of adolescents, particularly within disaster-prone regions.

Research shows that students' social resilience cannot be measured effectively through individual approaches alone; relational and contextual dimensions must be included. Adolescents, especially in disaster-prone areas, are strongly influenced by social interactions and the environment. This approach aligns with Bronfenbrenner's social ecology theory, which places individuals in a layered system ranging from family to public policy. The three-factor structure in SSRD (Individual, Relationship, and Contextual) is highly valid and reliable, with strong correlations between dimensions. This suggests that social resilience must be measured holistically and contextually. It can serve as a measurement tool and as a basis for designing educational interventions and policies that address the needs of students in disaster-affected areas.

This research builds on previous studies by recognizing that social resilience is multidimensional and offering a new approach that considers the local context of students in disaster-prone areas. This is important because, although most previous studies, such as those by Ungar [6] and Shi et al. [7], emphasized the interaction between individuals and the social environment, few have developed measurement instruments specifically designed for adolescents in areas with sociocultural characteristics and disaster risks, such as Indonesia. Through EFA and CFA, this study proves that the three dimensions (Individual, Relationship, and Contextual) consistently emerge with high validity and reliability. Therefore, this study's novel contribution is integrating local sociocultural dimensions into the SSRD instrument, making it a relevant and applicable measurement tool for disaster education in Indonesia.

The results of CFA indicate that each indicator in the SSRD demonstrated significant factor loadings and fell into the moderate to high category (> 0.50). This finding suggests a substantial contribution to the measured construct [32]. The Individual Factor encompasses items that delineate students' personal aptitude for emotion management, motivation maintenance, and adaptation strategy development. These items are congruent with the dimensions of personal resilience as delineated in the extant literature [29]. The Relationship Factor is indicative of the social support received from peers, teachers, and family members. This social support has been proven to be a strong predictor of psychosocial recovery in the aftermath of a disaster [14]. The Contextual Factor encompasses items pertaining to community involvement, access to resources, and institutional support, which are pivotal in fostering community-based social resilience [20]. The synergistic contributions of these three factors serve to enhance the construct validity of the SSRD as an instrument capable of comprehensively capturing students' social resilience.

The subsequent phase entails the incorporation of the SSRD instrument into educational policies and practices in schools susceptible to disasters. This is of particular importance because a valid and contextualized measurement of social resilience can serve as the foundation for designing psychosocial interventions and learning programs that are responsive to students' needs. Preliminary research findings indicate that the SSRD demonstrates high validity and reliability in measuring three primary dimensions of student resilience: individual, social relationships, and environmental context. The systematic implementation of SSRD in educational institutions will facilitate the identification of vulnerable students by teachers, counselors, and policymakers, thereby enabling the development of targeted resilience-strengthening programs. Consequently, the extensive implementation of SSRD can serve as a strategic measure in the development of a disaster-resilient education system that prioritizes not only academic components but also students' comprehensive social resilience.

## Conclusion

The present study successfully developed and validated the SSRD as an instrument for measuring student social resilience in disaster-prone areas. The results of the EFA and CFA indicate that the SSRD comprises three primary dimensions: Individual, Relationship, and Contextual. These dimensions exhibit robust validity and reliability. The Individual dimension is identified as a second-order construct that includes three sub-indicators: The following three factors have been identified as key elements in broadening the understanding of students' personal capacity in dealing with disasters: self-efficacy and emotional regulation, problem-solving and adaptability, and motivation and perseverance. All dimensions demonstrate a substantial contribution to the construct of social resilience, as evidenced by adequate factor loadings and optimal model fit. Consequently, SSRD can be utilised as an accurate and comprehensive measurement tool to identify students' levels of social resilience, while supporting educational interventions and policies that are responsive to the needs of disaster-prone areas.

This research makes a significant contribution to the development of knowledge in the field of educational psychology and disaster, especially in the context of measuring adolescent social resilience. SSRD has emerged as a novel instrument that integrates a multidimensional and local context-based approach, a methodology that was previously infrequently documented in the resilience literature. This instrument addresses a critical gap in existing research, which has historically prioritized either the individual or community dimensions in isolation. By drawing on social ecology theory and a multisystem approach, the SSRD not only supports theoretical understanding of resilience, but also provides a practical basis for developing intervention programs in schools. Consequently, SSRD can be utilized as a pertinent model instrument for subsequent research and implementation of disaster resilience education policies in various regions exhibiting analogous characteristics.

While the results of this study demonstrate considerable promise, there is room for further development. The present study focused on secondary school students; therefore, the application of SSRD to a broader population or across regions still requires additional verification. Furthermore, the instrument's sensitivity to temporal changes or post-intervention alterations has not been thoroughly evaluated through sensitivity testing, thereby leaving avenues for longitudinal research uncharted. The variation in contributions among indicators in the model provides valuable insights for the enhancement and enrichment of SSRD items in the future. Furthermore, cross-cultural testing across various social and educational contexts will enhance the external validity of SSRD, rendering it a more robust tool for measuring social resilience in diverse disaster situations..

## Data Availability

The datasets generated and analyzed during the current study are available from the corresponding author on reasonable request.

## Abbreviations

CFA: Confirmatory factor analysis
CFI: Comparative fit index
CYRM-28: Children and youth resilience measure
EFA: Exploratory factor analysis
GFI: Goodness of fit index
IM&P: Motivation & perseverance
IP&A: Problem-solving & adaptability
IS&ER: Self-efficacy & emotional regulation
KMO: Kaiser-Meyer-Olkin
MSA: Measure of sampling adequacy
MSPSS: Multidimensional scale of perceived social support
RMSEA: Root mean square error of approximation
SSRD: Student social resilience scale for disasters
TLI: Tucker-Lewis index
